# Smooth muscle Acid-sensing ion channel 1a as a therapeutic target to reverse hypoxic pulmonary hypertension

**DOI:** 10.3389/fmolb.2022.989809

**Published:** 2022-10-05

**Authors:** Selina M. Garcia, Tracy R. Yellowhair, Neil D. Detweiler, Rosstin Ahmadian, Lindsay M. Herbert, Laura V. Gonzalez Bosc, Thomas C. Resta, Nikki L. Jernigan

**Affiliations:** Department of Cell Biology and Physiology, University of New Mexico School of Medicine, Albuquerque, NM, United States

**Keywords:** vascular remodeling, endothelium, proliferation, migration, right heart hypertrophy

## Abstract

Acid-sensing ion channel 1a (ASIC1a) is a voltage-independent, non-selective cation channel that conducts both Na^+^ and Ca^2+^. Activation of ASIC1a elicits plasma membrane depolarization and stimulates intracellular Ca^2+^-dependent signaling pathways in multiple cell types, including vascular smooth muscle (SM) and endothelial cells (ECs). Previous studies have shown that increases in pulmonary vascular resistance accompanying chronic hypoxia (CH)-induced pulmonary hypertension requires ASIC1a to elicit enhanced pulmonary vasoconstriction and vascular remodeling. Both SM and EC dysfunction drive these processes; however, the involvement of ASIC1a within these different cell types is unknown. Using the Cre-LoxP system to generate cell-type-specific *Asic1a* knockout mice, we tested the hypothesis that SM-*Asic1a* contributes to CH-induced pulmonary hypertension and vascular remodeling, whereas EC-*Asic1a* opposes the development of CH-induced pulmonary hypertension. The severity of pulmonary hypertension was not altered in mice with specific deletion of EC-*Asic1a* (Tek^Cre^-*Asic1a* ^fl/fl^). However, similar to global *Asic1a* knockout (*Asic1a* ^−/-^) mice, mice with specific deletion of SM-*Asic1a* (MHC^CreER^-*Asic1a* ^fl/fl^) were protected from the development of CH-induced pulmonary hypertension and right heart hypertrophy. Furthermore, pulmonary hypertension was reversed when deletion of SM-*Asic1a* was initiated in conditional MHC^CreER^-*Asic1a* ^fl/fl^ mice with established pulmonary hypertension. CH-induced vascular remodeling was also significantly attenuated in pulmonary arteries from MHC^CreER^-*Asic1a* ^fl/fl^ mice. These findings were additionally supported by decreased CH-induced proliferation and migration of pulmonary arterial smooth muscle cells (PASMCs) from *Asic1a* ^−/-^ mice. Together these data demonstrate that SM-, but not EC-*Asic1a* contributes to CH-induced pulmonary hypertension and vascular remodeling. Furthermore, these studies provide evidence for the therapeutic potential of ASIC1a inhibition to reverse pulmonary hypertension.

## 1 Introduction

Under normal physiological conditions, the pulmonary circulation is maintained in a low-pressure, low-resistance state, with little or no resting vascular tone. During pathological conditions, a sustained increase in pulmonary vascular resistance leads to the development of pulmonary hypertension. Pulmonary hypertension is a progressive and often fatal pulmonary vascular disease defined by a mean pulmonary arterial pressure >20 mmHg (Simonneau et al., 2019). Over time, the elevated vascular resistance and pulmonary arterial pressure increase right ventricular afterload. When the adaptive mechanisms of right ventricular dilation and hypertrophy can no longer compensate for the high vascular resistance in the lung, right heart failure occurs and is associated with a poor prognosis.

Although pulmonary hypertension stems from different underlying causes, the increase in pulmonary vascular resistance in all forms of pulmonary hypertension can be attributed to a combination of sustained pulmonary vasoconstriction and vascular remodeling. Enhanced vasoconstriction is linked to pulmonary arterial endothelial cell (PAEC) dysfunction and hyperreactivity of pulmonary arterial smooth muscle cells (PASMCs) (Budhiraja et al., 2004; Lin et al., 2004; Nagaoka et al., 2004; Jernigan et al., 2008; Broughton et al., 2010; Weise-Cross et al., 2018). The impact of remodeling on pulmonary vascular resistance is primarily due to thickening of the intimal and/or medial layer of small muscular arteries and distal neomuscularization, depicted by the appearance of cells expressing smooth muscle (SM)-specific markers in normally non-muscular precapillary, intra-acinar vessels. This complex pathogenesis is thought to be initiated by endothelial cell (EC) injury and apoptosis followed by the emergence of excessive proliferation and migration of apoptosis-resistant PAECs and PASMCs, and cellular trans-differentiation in the form of EC-mesenchymal transition and SM phenotypic transformations (Voelkel & Tuder, 2000; Shimoda & Laurie, 2013; Gao et al., 2016). Metabolic derangements that promote aerobic glycolysis and inhibition of mitochondrial oxidative respiration have been shown to drive the extensive right ventricular and vascular remodeling in both animal models and patients with pulmonary hypertension (McMurtry et al., 2004; Bonnet et al., 2006; Xu et al., 2007; Sutendra et al., 2010; Fessel et al., 2012; Dromparis et al., 2013; Pak et al., 2013). The shift in cellular metabolism to lactic acid fermentation leads to pathological increases in extracellular acidity. Several ion channels are either directly gated or their activity modulated by alterations in intracellular and extracellular pH including acid-sensing ion channels (ASIC), transient receptor potential vanilloid receptor 1 (TRPV1), the transient receptor potential ankyrin repeat receptor 1 (TRPA1), some two-pore domain (K2P) channels, inwardly rectifying K^+^ channels (Kir), and voltage-gated Na^+^, Ca^2+^, and K^+^ channels (Harguindey et al., 2017).

Acid-sensing ion channels (ASICs) constitute a subfamily of the amiloride-sensitive, degenerin/epithelial Na^+^ channel (Deg/ENaC) superfamily that form H^+^-gated, voltage-insensitive cation channels. Similar to ENaCs, ASICs are highly selective for Na^+^ over other ions; except ASIC1a which additionally conducts Ca^2+^ (Waldmann et al., 1997; Xiong et al., 2004; Yermolaieva et al., 2004). The influx of Na^+^ and Ca^2+^ contributes to membrane depolarization, activation of Ca^2+^–calmodulin-dependent mechanisms, and other second-messenger pathways signifying the diverse roles played by ASIC1a in intracellular signaling and excitability under both normal and pathological conditions. ASICs have been primarily studied in neurons due to their ubiquitous expression throughout the central and peripheral nervous systems. Consequently, it is less well recognized that ASICs are expressed in a variety of other cell types including oligodendrocytes, mesenchymal, epithelial, endothelial, muscle, adipose/endocrine, and immune cells where they have been implicated in a range of pathologies (Foster et al., 2021; Karlsson et al., 2021). Although the expression of ASIC1 has been reported in vascular SM and ECs (Grifoni et al., 2008; Jernigan et al., 2009; Chung et al., 2010; Akanji et al., 2019; Garcia et al., 2020; Redd et al., 2021), less is known about the functional role of ASIC1 to regulate vascular homeostasis in disease states.

Previous studies from our laboratory have identified a novel role for ASIC1a in the development of chronic hypoxia (CH)-induced pulmonary hypertension (Nitta et al., 2014). ASIC1 is expressed in both PASMCs and PAECs; however, the contribution of ASIC1a to the pathological mechanisms leading to pulmonary artery remodeling and the development of pulmonary hypertension in these two vascular cell types is unclear. While prior studies indicate PASMC ASIC1a mediates pulmonary vasoconstriction (Jernigan et al., 2012), the functional role of ASIC1 in PAECs is unknown. Based on studies showing EC ASIC1 in mesenteric arteries contributes to endothelial-dependent vasodilation (Garcia et al., 2018), we speculate PAEC ASIC1a may be protective against the development of pulmonary hypertension. To test the hypotheses that SM-*Asic1a* contributes to CH-induced pulmonary hypertension and vascular remodeling and EC-*Asic1a* opposes the development of CH-induced pulmonary hypertension we will use the Cre-loxP system to generate mice with selective EC-*Asic1a* deletion (Tek^Cre^-*Asic1a* ^fl/fl^) or inducible SM-*Asic1a* deletion (MHC^CreER^-*Asic1a* ^fl/fl^).

## Materials and methods

### Ethical approval

All protocols used in this study were reviewed and approved by the Institutional Animal Care and Use Committee of the University of New Mexico School of Medicine (Protocol #19-200899-HSC) and abide by the National Institutes of Health guidelines for animal use. All animals were anesthetized with an overdose of pentobarbital sodium (200 mg/kg, i.p.) and immediately euthanized by exsanguination after the loss of consciousness.

### Animals

Studies were completed in adult male wildtype (*Asic1a* ^+/+^) or various transgenic mice (12–16 weeks old) as shown in Table 1. To selectively delete *Asic1a* in ECs or SM, *Asic1a* ^fl/fl^ mice were crossed with Tek^Cre^ or MHC^CreER^ transgenic mice, respectively. Homozygote and/or heterozygote mice were bred and Cre transgene expression and deletion of the *Asic1a* gene were confirmed by PCR and agarose gel electrophoresis (Table 1). Animals were housed one to five per cage in a specific pathogen-free (SPF) animal care facility and maintained on a 12:12-h light-dark cycle. Standard chow (Teklad soy protein-free diet #2920, Envigo) and water were provided *ad libitum*. Animals were randomly allocated to experimental groups and when possible, genotype and treatment assignments were blinded to the investigators. Male mice were studied exclusively since the expression of iCreER^T2^ under the control of the SM promoter is inserted on the Y chromosome. Furthermore, our previous data showed no significant interaction between sex and the development of hypoxic pulmonary hypertension (Nitta et al., 2014). For induction of Cre activity, MHC^CreER^-*Asic1a* ^fl/fl^ mice were injected with 75 mg/kg tamoxifen (TAM; Sigma-Aldrich, CAS #10540-29–1) in corn oil, once daily for five consecutive days. Following 14 days, Cre recombinase was assessed from tail DNA using the 5′ LoxP forward and 3′ LoxP reverse primer pair (Table 1). Tamoxifen was administered to MHC^CreER^-*Asic1a* ^fl/fl^ mice to induce SM-*Asic1a* knockout either 1) 2 weeks before CH as a preventative (pTAM) protocol or 2) following 3 weeks CH as a therapeutic (tTAM) protocol to assess reversal of established pulmonary hypertension. We have previously shown that mice develop pulmonary hypertension following 3-weeks CH exposure (Sheak et al., 2020). *Asic1a* disruption was assessed from total RNA (1 µg) in the brain (positive control) and isolated pulmonary arteries by RT-PCR. Total RNA was extracted using TRIzol and reversed transcribed to cDNA (Transcription First-Strand cDNA Synthesis kit, Roche, 04379012001). Amplification of *Asic1a* was achieved by PCR (iCycler, Bio-Rad) using REDExtract-N_Amp PCR Ready Mix (Sigma-Aldrich, XNAT) and *Asic1* primers: forward: 5′ CAC​ATG​CCA​GGG​GAT​GCC​CC 3′ and reverse: 5′ AGC​CGG​TGC​TTA​ATG​ACC​TC 3’ (410 bp). The PCR product was separated using gel electrophoresis on a 3% agarose gel and stained with ethidium bromide for visualization under UV light.

**TABLE 1:** Transgenic mouse models, source, reference, primers, and expected base pairs to identify each genotype.

Transgenic model, source, RRID#	Ref	Genotyping primers 5’ → 3′	bp
***Asic1a*** ^**−/−**^ (B6.129-*Asic1* ^tm1Wsh^/J) The Jackson Laboratory RRID #: IMSR_JAX:013733	(Wemmie et al., 2002)	***Asic1*** ^**+/+**^ **forward**: CATGTCACCAAGCTCGACGAGGTG ***Asic1*** ^**−/−**^ **forward**: TGGATGTGGAATGTGTGCGA ^**+/+**^ **and** ^**−/−**^ **reverse**: CCGCCTTGAGCGGCAGGTTTAAAGG	262 310
***Asic1a*** ^**fl/fl**^ (B6.129-*Asic1* ^tm1Lien^) National Laboratory Animal CenterRMRC #: 13158	Wu et al. (2013)	**5′LoxP forward**: TCCTCTCCCAAACACACAC **5′LoxP reverse**: GAGTTCCCTCCAGATGTGAG **3′ LoxP forward**: AGGCCTGCAAACTGTCATCT **3′ LoxP reverse**: GTTGCATCTTGAGCCTCCTC	410
			406
**Tek** ^**Cre**^ (B6.Cg-Tg (Tek-cre)^1Ywa^/J) The Jackson Laboratory RRID #: IMSR_JAX:008863	Kisanuki et al. (2001)	**Cre Forward**: GCGGTCTGGCAGTAAAAACTATC **Cre Reverse**: GTGAAACAGCATTGCTGTCACTT	100
**MHC** ^**CreER**^ (B6.FVB-Tg (Myh11-icre/ERT2)1Soff/J) The Jackson Laboratory RRID: IMSR_JAX:019079	Wirth et al. (2008)	**Cre Forward**: TGACCCCATCTCTTCACTCC **Cre Reverse**: AGTCCCTCACATCCTCAGGTT	287

#### Assessment of Systemic Mean arterial Blood Pressure

Blood pressure and heart rate were recorded in mice using radiotelemetry devices (PA-C10 implant; Data Systems International). Telemetry transmitters were surgically implanted under sterile conditions with inhaled isoflurane anesthesia (2% isoflurane and 98% O_2_ gas mixture). The analgesic Buprenex (buprenorphine; 0.1 mg/kg, IM) was administered before the start of surgery to provide effective recovery and preemptive pain management. Using sterile techniques, a midline incision was made to expose the carotid artery by blunt dissection. A small incision was made in the carotid artery between two silk sutures and the end of the catheter of a PA-C10 small implantable telemetry probe was inserted and advanced toward the heart. The tip was tied in place and the body of the telemeter was secured subcutaneously in the mid-flank region of the carotid artery. The wound was closed with sterile suture and the mouse was allowed to recover 5 days before blood pressure measurements. Blood pressure was recorded for 72 h (every 15 min for 10s-intervals) and data were presented as 24 h averages

#### Exposure to CH

CH is a common complication of chronic lung diseases and a key stimulus in the development of pulmonary hypertension. Animals designated for exposure to CH were housed in a clear-plexiglass hypobaric chamber (∼0.5 m^3^) with barometric pressure maintained at ∼380 mmHg for 6 weeks. The hypobaric chamber was partially evacuated with a vacuum pump allowing for continuous airflow of 30 L/min through the chamber. The chamber was opened 2 times a week to change bedding and provide fresh water and food. Age-matched control animals were housed at ambient barometric pressure (∼630 mmHg in Albuquerque, NM). We have previously demonstrated that this mouse model mimics many of the cardiopulmonary changes observed in human pulmonary hypertension including increased right ventricular systolic pressure, right ventricular hypertrophy, enhanced vasoconstriction, and arterial remodeling (Nitta et al., 2014; Detweiler et al., 2019; Sheak et al., 2020).

### Immunofluorescence from paraffin-embedded lung tissue

Mice were anesthetized with pentobarbital sodium (200 mg/kg i. p.). After a median sternotomy, heparin (100 U/20 g body wt) was injected directly into the RV, and the pulmonary artery was cannulated with a 22-gauge feeding needle. The preparation was immediately perfused with 0.1 M PBS containing 10^–4^ M papaverine to maximally dilate the vasculature and flush the circulation of blood. The lungs were then perfused with 25 ml fixative (0.1 M PBS containing 4% sucrose, 4% paraformaldehyde, and 10^–4^ M papaverine) at a pressure of 50 cm H_2_O above the hilum, and the trachea inflated to a pressure of 25 cm H_2_O creating a transmural distending pressure of 25 cm H_2_O during fixation to ensure vessels were fully dilated. The trachea was ligated with 4–0 silk, and the lungs were immersed in fixative overnight, dehydrated, and then mounted in paraffin.

Sections were cut (5 μm thick) and mounted onto Superfrost Plus slides (Fisher Scientific). Antibody-antigen binding was enhanced by heat-mediated antigen retrieval using either Tris-EDTA Buffer (10 mM Tris, 1 mM EDTA, 0.05% Tween-20, pH 9) for 15 min at 100°C (for Ki-67) or Citric-Acid-Sodium Citrate Buffer (pH 6, 0.05% Tween-20) for 25 min at 100°C (for remodeling and ASIC1 expression). Sections were incubated with primary (24 h at 4°C) and secondary antibodies (24 h at 4°C) as indicated in Table 2. We have previously determined the specificity of goat anti-ASIC1 using wild-type and knockout mice (Nitta et al., 2014). Sections were mounted with FluoroGel (Electron Microscopy Sciences), and cross-section images of pulmonary arterioles (<100 µm) were acquired sequentially by confocal microscopy (TCS SP5, Leica) using Argon (488 nm/∼20 mW, HeNe (543 nm/∼1 mW), and HeNe (633 nm/∼10 mW) class IIIb lasers and a ×63 objective.

**TABLE 2:** List of primary and secondary antibodies used for immunofluorescence and western blot analysis.

Antibody	Company	Cat #	RRID	Host; Clone	Dilution	Figures
PRIMARY						
anti-ki-67 (SP6)	Thermo Fisher Scientific	RM-9106	AB_2341197	rabbit; mono	1:300	1, 6
anti-ki-67 (B56)	BD Biosciences	550609	AB_393778	mouse; mono	1:100	1
anti-actin, α-SM	Sigma-Aldrich	A2547	AB_476701	mouse; mono	1:300	1, 3, 5, 6
anti-CD31	Abcam	ab124432	AB_2802125	rabbit; poly	1:200	1, 3
anti-SMMHC II	Biomedical Technologies	BT-562	AB_10013421	rabbit; poly	1:1,000	2
anti-GAPDH	Sigma-Aldrich	G9545	AB_796208	rabbit; poly	1:1,000	2
anti-ASIC1 (E-15)	Santa Cruz Biotechnology	sc-13903	AB_633515	goat; poly	1:50	3
anti-ASIC1	Millipore-Sigma	AB5674P	AB_91972	Rabbit; poly	1:500	8
anti-CD31	Abcam	ab124432	AB_2802125	rabbit; poly	1:200	3
SECONDARY						
Alexa Fluor^®^ 488 Anti-Rabbit IgG (H + L)	Jackson ImmunoResearch Laboratories, Inc.	711-546-152	AB_2340619	donkey; poly	1:500	3
Alexa Fluor^®^ 488 Anti-Mouse IgG (H + L)	Jackson ImmunoResearch Laboratories, Inc.	715-546-150	AB_2340849	donkey; poly	1:500	1, 5, 6
Cy™3 AffiniPure Anti-Goat IgG (H + L)	Jackson ImmunoResearch Laboratories, Inc.	705-165-147	AB_2307351	donkey; poly	1:500	3
Cyanine Cy™3 Anti-Rabbit IgG (H + L)	Jackson ImmunoResearch Laboratories, Inc.	711-165-152	AB_2307443	donkey; poly	1:500	1, 6
Alexa Fluor^®^ 647 Anti-Mouse IgG (H + L)	Jackson ImmunoResearch Laboratories, Inc.	715-605-150	AB_2340862	donkey; poly	1:500	3
Anti-Rabbit IgG (H + L)-HRP Conjugate	Bio-Rad	1721019	AB_11125143	goat; poly	1:3,000	2

#### Assessment of cellular proliferation using Ki-67

Lung sections were incubated with anti-Ki-67 (Table 2) and the percent Ki-67 positive SM and ECs were calculated from ∼15-20 vessels per animal (5 animals/group) using ImageJ software (National Institutes of Health). Vessels were identified by morphology and SMA or CD31 immunofluorescence and nuclei were stained with TO-PRO™-3 iodide (1:1,000; Invitrogen, T3605) for 15 min at room temperature before mounting the sections.

#### Assessment of cell-specific ASIC1 deletion

Lung sections were incubated with antibodies against ASIC1, SMA, and CD31 (Table 2). Images were taken of five pulmonary arteries per group. Using ImageJ software (NIH), a mask was made of either SMA or CD31 immunofluorescence and the mean intensity of ASIC1 was determined in each mask.

#### Assessment of arterial remodeling using α-SM actin immunofluorescence

Images were thresholded using ImageJ software. Regions of interest (ROIs) were drawn around each fully muscularized artery. The percent thresholded area to total ROI area was calculated for each artery and multiplied by 100 to get the percent muscularization. Arterial diameter was calculated based on the circumference of the ROI and analysis was conducted by arterial diameter: <25 μm, 25–50 μm, or 50–100 μm. Fluorescence images were digitally inverted to provide better contrast and visibility of immunofluorescence.

### Western blot analysis

SM myosin heavy chain (MHC) and GAPDH protein expression were determined by western blot analysis. The whole lung was homogenized in Tris-HCl homogenization buffer (containing 225 mM sucrose, 2 mM Tris-HCL, 2 mM EDTA, 12 µM leupeptin, 1 µM pepstatin A, and 0.3 µM aprotinin) with a glass homogenizer and centrifuged at 10,000 g for 10 min at 4°C to remove insoluble debris. Sample protein concentrations were determined by the Qubit Protein Assay (Life Technologies). Samples were boiled for 5 min in sample buffer and 20 µg of protein was separated by SDS-PAGE (7.5% Tris/glycine) and transferred to a polyvinylidene difluoride membrane. The blot was blocked at room temperature for 1 h with 5% nonfat dry milk then incubated overnight at 4°C in primary antibodies followed by 1 h at room temperature in secondary antibodies (Table 2). Proteins were then detected by autoradiography film (GeneMate) following chemiluminescence labeling (ECL; Pierce, 32209). Quantification of protein expression was done using ImageJ software and MHC expression was normalized to GAPDH. GAPDH was probed subsequently to MHC.

### Assessment of pulmonary hypertension

Following 6 weeks CH, mice were anesthetized (2% isoflurane and 98% O_2_ gas mixture) and right ventricular systolic pressure (RVSP) and heart rate were measured via transdiaphragmatic direct cardiac puncture as previously described (Nitta et al., 2014). An upper transverse laparotomy was performed to expose the diaphragm. A 25-gauge needle, connected to a pressure transducer (model APT300, Harvard Apparatus) through a saline-filled catheter, was inserted into the RV via a closed-chest transdiaphragmatic approach, and the output amplified using a TAM-A bridge amplifier (Hugo Saks Electronik; Harvard Apparatus) and recorded using Powerlab data acquisition and LabChart software (ADInstruments). The derivative of max RV pressure over time (dP/dt_max_) provides an index of RV contractility. The pressure-time index is the area under the systolic pressure curve and is indicative of RV workload and oxygen consumption. Right ventricular hypertrophy in response to CH was assessed by measuring the mass ratio of the right ventricle to left ventricle plus septum (Fulton’s index).

### Proliferation/migration in PASMCs

#### Generation of mouse PASMCs (mPASMC)

Animals were anesthetized with pentobarbital sodium (200 mg/kg body weight, IP), and the heart and lungs were removed by midline thoracotomy. Intrapulmonary arteries (∼second–fifth order) were dissected from surrounding lung parenchyma and enzymatically digested by incubating in reduced-Ca^2+^ Hank’s Balanced Salt Solution (HBSS) containing papain (9.5 U/ml), type-I collagenase (2 mg/ml), dithiothreitol (1 mg/ml), and BSA (2 mg/ml) at 37°C for 20 min. PASMCs were dispersed by gentle trituration with a fire-polished pipette in Ca^2+^-free HBSS. Freshly dispersed PASMCs were plated on gelatin-coated dishes and cultured in SM Cell Medium (Cell Biologics) containing 10% fetal bovine serum and 1% penicillin/streptomycin in a humidified atmosphere of 5% CO_2_-95% air at 37°C. Before experiments, PASMCs were cultured for at least 48 h in a serum-free SMC Medium containing insulin, EGF, hydrocortisone, l-glutamine, and 1% penicillin/streptomycin (M2268SF Cell Biologics). Cellular purity was >95%, as assessed by morphological appearance under phase-contrast microscopy and immunofluorescence staining for SM 22α as previously described (Detweiler et al., 2019).

#### mPASMC Proliferation

To determine the involvement of ASIC1a in proliferation, mPASMCs from *Asic1a* ^+/+^ and *Asic1a* ^−/−^ mice were incubated with bromodeoxyuridine (BrdU; 10 μM) for 24, 48, and 72 h hypoxia (2% O_2_, 5% CO_2_) using a hypoxic incubator subchamber (Biospherix C-Chamber). mPASMCs were fixed and labeled with a conjugated Anti-BrdU FITC antibody (BD Biosciences) to measure BrdU incorporation in newly synthesized DNA of 20,000 events per sample by flow cytometric analysis (LSR-Fortessa flow cytometer with FACSDiva, version 3.0 software; BD Biosciences). PDGF-BB (20 ng/ml, Millipore) was added for 72 h to generate a positive staining control.

#### mPASMC migration

mPASMC migration was assessed using a modified Boyden chamber (Costar Transwell inserts 6.5 mm diameter, 8.0 µm pore size). mPASMCs were counted using a standard grid assay and plated on the insert at 1 × 10^5^ cells/well in basal media (plus 1% FBS). Basal media was also added to the lower well of the Boyden chamber and the cells were incubated 24 h in normoxia (95% air, 5% CO_2_) or hypoxia (2% O_2_, 5% CO_2_) to stimulate migration. After 24 h, mPASMCs were fixed with 2% paraformaldehyde for 15 min and then stained with Coomassie Blue for 5 min. Cells were washed several times to remove excess Coomassie and images were taken with a ×20 objective on an Eclipse E400 microscope with a DS-Fi1 camera and analyzed using NIS-Elements F 3.0 software (Nikon). Five random brightfield images were taken per well for the total number of cells before the un-migrated PASMCs from the top of the filters were removed with a cotton swab and an additional five images were taken to obtain the number of migrated cells. The Coomassie-stained mPASMCs were used to determine the area of migrated to total mPASMCs, which was multiplied by 100 to get the percent migrated mPASMC (ImageJ).

Human PASMCs (hPASMC; Cascade Biologics, #C-009-5C) were grown on poly-l-lysine-coated plates in Media 231 (Invitrogen, #M231500) with SM growth supplement (Invitrogen, #S00725) in a humidified atmosphere of 5% CO_2_-95% air at 37°C. hPASMC were used at passage three to five and were treated with vehicle (H_2_O), amiloride (30 μM, Enzo Life Sciences), or psalmotoxin 1 (PcTX1; 20 nM, Phoenix Pharmaceuticals) for the duration of the following experiments.

#### ASIC1 expression in hPASMC

Total and cell surface expression of ASIC1 was determined by western blot analysis (see above). hPASMC were grown until ∼90% confluent in 75-cm^2^ flasks and exposed to 12 h normoxia (95% air, 5% CO_2_) or hypoxia (2% O_2_, 5% CO_2_). To determine plasma membrane localization of ASIC1, we used a cell surface protein isolation kit (Pierce, Thermo Fisher Scientific) as previously described (Herbert et al., 2016, 2018). hPASMCs were incubated with Sulfo-NHS-SS-Biotin (Pierce) for 30 min at 4°C. The reaction was quenched and hPASMCs were harvested and lysed with 10 mM TrisHCl homogenization buffer and spun at 10,000 g for 2 min. The clarified supernatant was added to NeutrAvidin Agarose resin columns for 1 h a room temperature. The flow-through was collected as the cytosolic protein fraction, and surface protein was collected by elution with 5× sample buffer. We previously demonstrated the specificity of cell surface assay to fractionate cell surface vs intracellular proteins (Herbert et al., 2016). Surface protein (25 μl) or cytosolic protein lysates (20 μg) were separated by SDS-PAGE (7.5% Tris/glycine) and transferred to PVDF membranes. ASIC1 was detected in cell surface and cytosolic fractions by exposure of the blot to chemiluminescence-sensitive film (GeneMate). Quantification of ASIC1 bands was accomplished by densitometric analysis of scanned images (ImageJ) and expressed as the ratio of plasma membrane to cytosolic densitometric units.

#### hPASMC migration

An *in vitro* scratch assay was performed on confluent monolayers of hPASMCs. Monolayers were manually scraped with a 100 µL pipette tip and then gently washed twice with PBS to remove non-adherent cells. Images of the wounded area were captured immediately after the scratch (time zero) and following a 12 h exposure to normoxia (95% air, 5% CO_2_) or hypoxia (2% O_2_, 5% CO_2_). Images were taken with a ×20 objective on an Eclipse E400 microscope with a DS-Fi1 camera and analyzed using NIS-Elements F 3.0 software (Nikon). A grid attached to the bottom of the cell culture plate was used as a reference point to capture images of the same location at each time interval. The wounded area was determined using ImageJ (National Institutes of Health). Healing was quantified as % Reinvasion = (AreaI–AreaT)/AreaI × 100%, where: AreaI = Initial area, and AreaT = Area at time (T) 12 h after injury.

#### hPASMC proliferation

hPASMCs were trypsinized and the cell suspension was mixed with equal parts Trypan blue solution to a final concentration of 0.4% to assess cell viability. A homogenous mixture was loaded into a disposable chamber side and the cell number was determined using the Countess automated cell counter (Invitrogen).

### Statistics

All data are expressed as means ± standard error. Percentage data were converted to normal distributions by arcsine transforms before parametric analysis. Normal distribution was tested using the Shapiro-Wilks Normality Test (*p* > 0.05). Values of n and statistical tests are specified in the figure legends and were made using Prism 9 (GraphPad Software). A probability of ≤ 0.05 with a power level of 0.80 was accepted as statistically significant for all comparisons.

## Results

### CH-induced vascular cell proliferation and phenotypic switch is ASIC1a dependent

**FIGURE 1:**
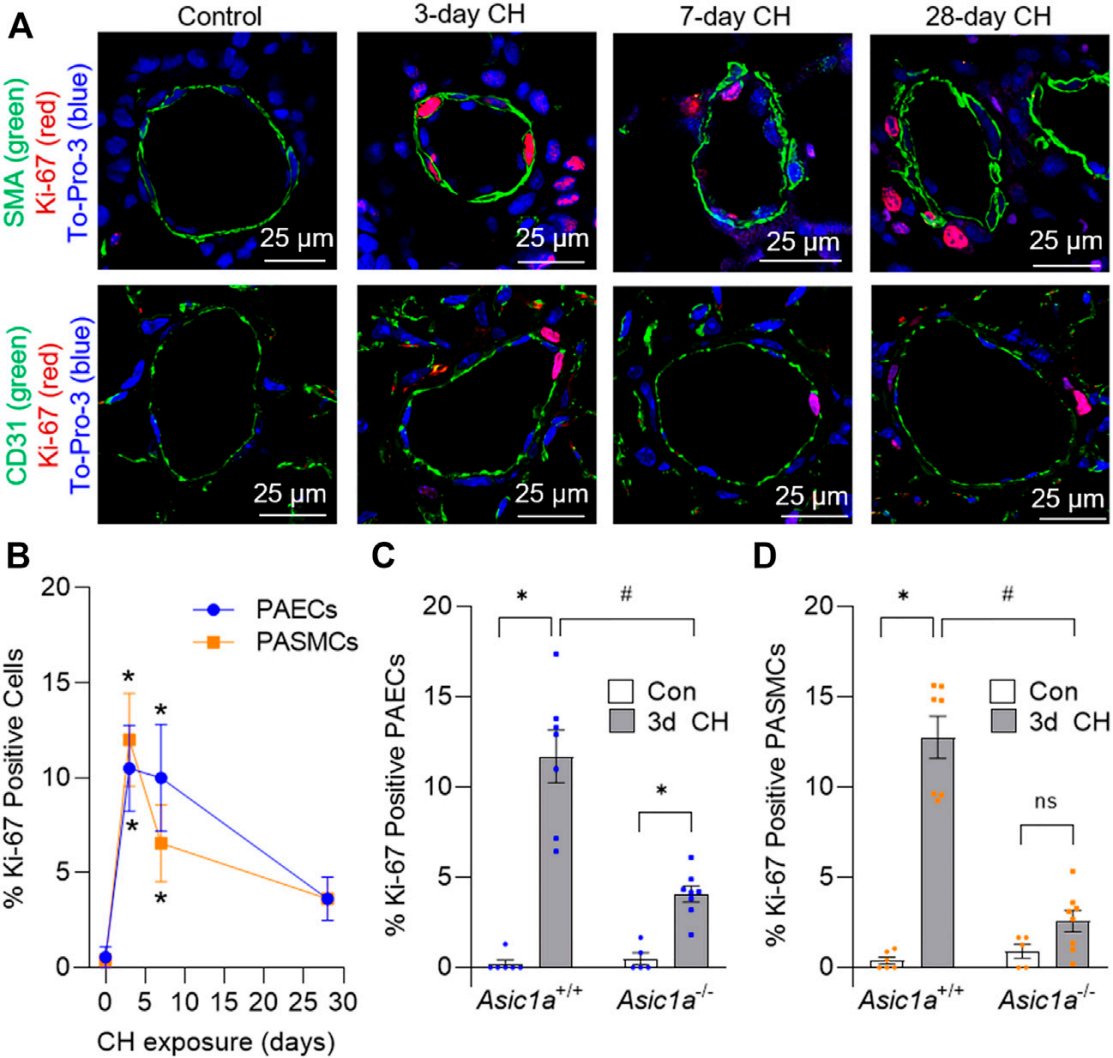
CH-induced proliferation of vascular cells is ASIC1a dependent. **(A)** Representative immunofluorescence images showing Ki-67 (red), α-SMA (green, top row), CD31 (green, bottom row), and To-Pro-3 (blue) in small pulmonary arteries (<100 µm) from *Asic1a* ^+/+^ mice and **(B)** summary data showing the percent Ki-67 positive pulmonary arterial endothelial cells (PAECs, blue circles) and pulmonary arterial smooth muscle cells (PASMCs, orange squares) under control conditions or following exposure to CH (3-, 7-, or 28-days). n = 3 animals per group (∼20 vessels were averaged for each animal); analyzed as one-way ANOVA for each cell type and individual groups compared with Šídák’s multiple comparisons tests. Summary data showing percent Ki-67 positive **(C)** PAECs and **(D)** PASMCs in small pulmonary arteries (<100 µm) from *Asic1a* ^+/+^ and *Asic1a* ^−/-^ mice under control conditions or following 3-days CH exposure. n = five to eight animals (∼15 vessels were averaged for each animal); analyzed by two-way ANOVA. Significant interactions between the individual groups (*p* < 0.0001 for both PAECs and PASMCs) were compared with Šídák’s multiple comparisons tests; *p < 0.05 vs. control; #*p* < 0.05 vs. corresponding 3-days CH; ns = not significant.

Ki-67 is a nuclear protein that is expressed during cellular proliferation. To examine *in vivo* proliferation of vascular cells over the development of CH-induced pulmonary hypertension we determined the percent Ki-67 positive PAECs and PASMCs cells following a 0-, 3-, 7-, and 28-day exposure to CH in *Asic1a* ^+/+^ mice (Figure 1A). The number of proliferating PAECs and PASMCs was highest following 3 days CH (Figure 1B). The percent of proliferating PAECs and PASMCs was still elevated by 7 days, but PASMC proliferation decreased by half. The percent proliferating PAECs and PASMCs at 28 days was not significantly different compared to baseline (Figure 1B), as the majority of proliferating cells at 28 days were extravascular cells. Based on these data, we then examined PAEC and PASMC proliferation in *Asic1a* ^−/-^ mice following 3 days CH. The percent of proliferating PAECs was significantly reduced in *Asic1a* ^−/-^ mice but was elevated compared to controls (Figure 1C). Moreover, there was no effect of CH to induce proliferation of PASMCs in *Asic1a* ^−/-^ mice (Figure 1D).

**FIGURE 2:**
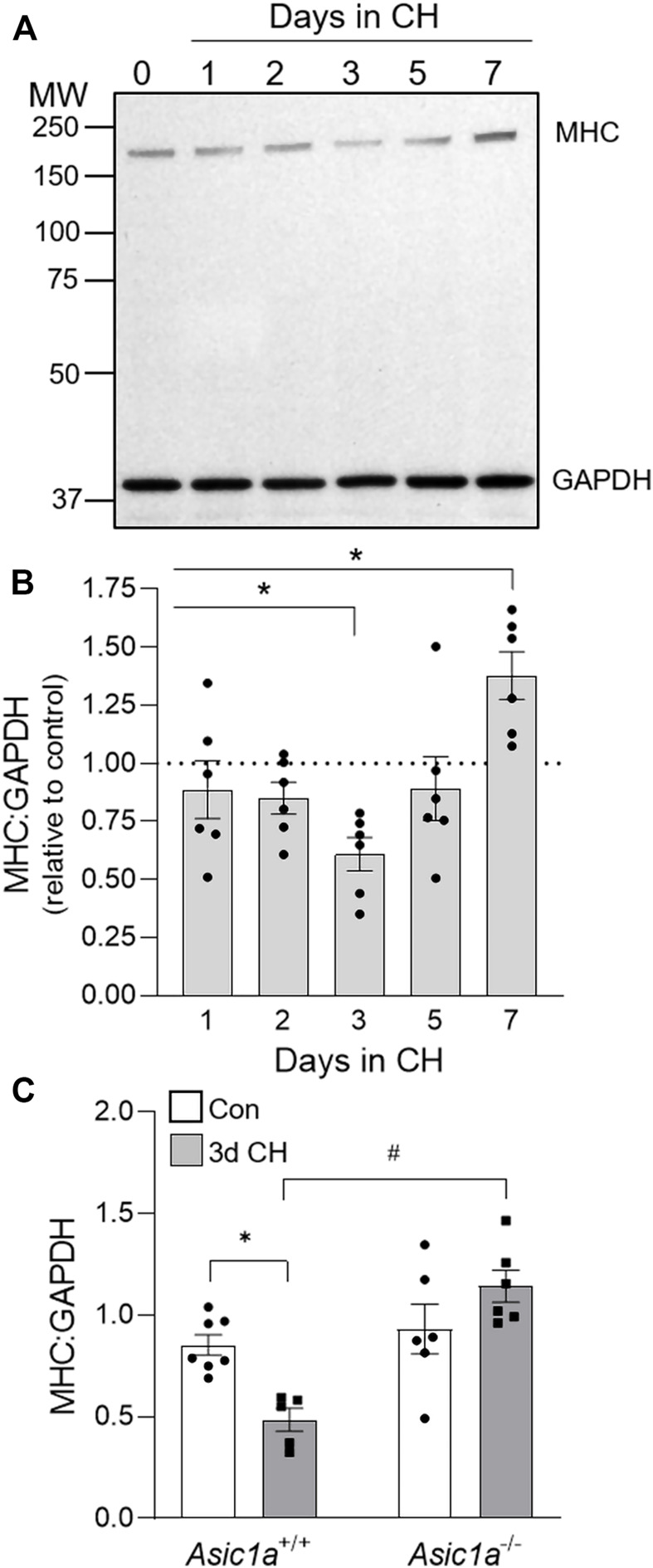
CH-induced loss of contractile protein, MHC, is ASIC1a dependent. **(A)** Representative western blot and **(B)** the effect of CH exposure (1–7 days) on MHC to GAPDH protein expression in whole lung homogenates in *Asic1a* ^*+/+*^ animals. The scanned image of the film was converted to greyscale and adjusted for brightness/contrast. n = 6/group; analyzed by one-way ANOVA and individual groups compared with Šídák’s multiple comparisons tests. **(C)** Summary data for MHC to GAPDH in whole lungs from *Asic1a* ^+/+^ and *Asic1a* ^−/-^ mice under control conditions or following 3-days CH exposure. n = 5-7 animals/group; analyzed by two-way ANOVA. Significant interactions between the individual groups (*p* = 0.0022) were compared with the Šídák’s multiple comparisons tests; *p < 0.05 vs. control; #*p* < 0.05 vs. *Asic1a* ^+/+^ mice.

We next determined if the CH-induced increase in PASMC proliferation is associated with a loss in contractile phenotype by analyzing the protein expression of SM myosin heavy chain (MHC) in lung tissue at 0- (Con), 1-, 2-, 3-, 5-, and 7-days CH (Figure 2A). Consistent with the greatest percent of Ki-67 positive PASMCs following 3 days CH (Figure 1B), MHC was significantly decreased after 3-days CH in *Asic1a* ^*+/+*^ animals (Figures 2B,C). After 7 days of CH, MHC was increased compared to control levels (Figure 2B). CH did not decrease MHC levels in *Asic1a* ^−/-^ mice (Figure 2C). Hypoxia did not change expression levels of GAPDH (*p* = 0.2082). Together, these data suggest ASIC1a contributes to PAEC and PASMC proliferation and PASMC phenotypic change that is seen in pulmonary arteries in response to CH exposure.

### SM-specific knockout of *Asic1a* protects against the development of and reverses hypoxic pulmonary hypertension

**FIGURE 3:**
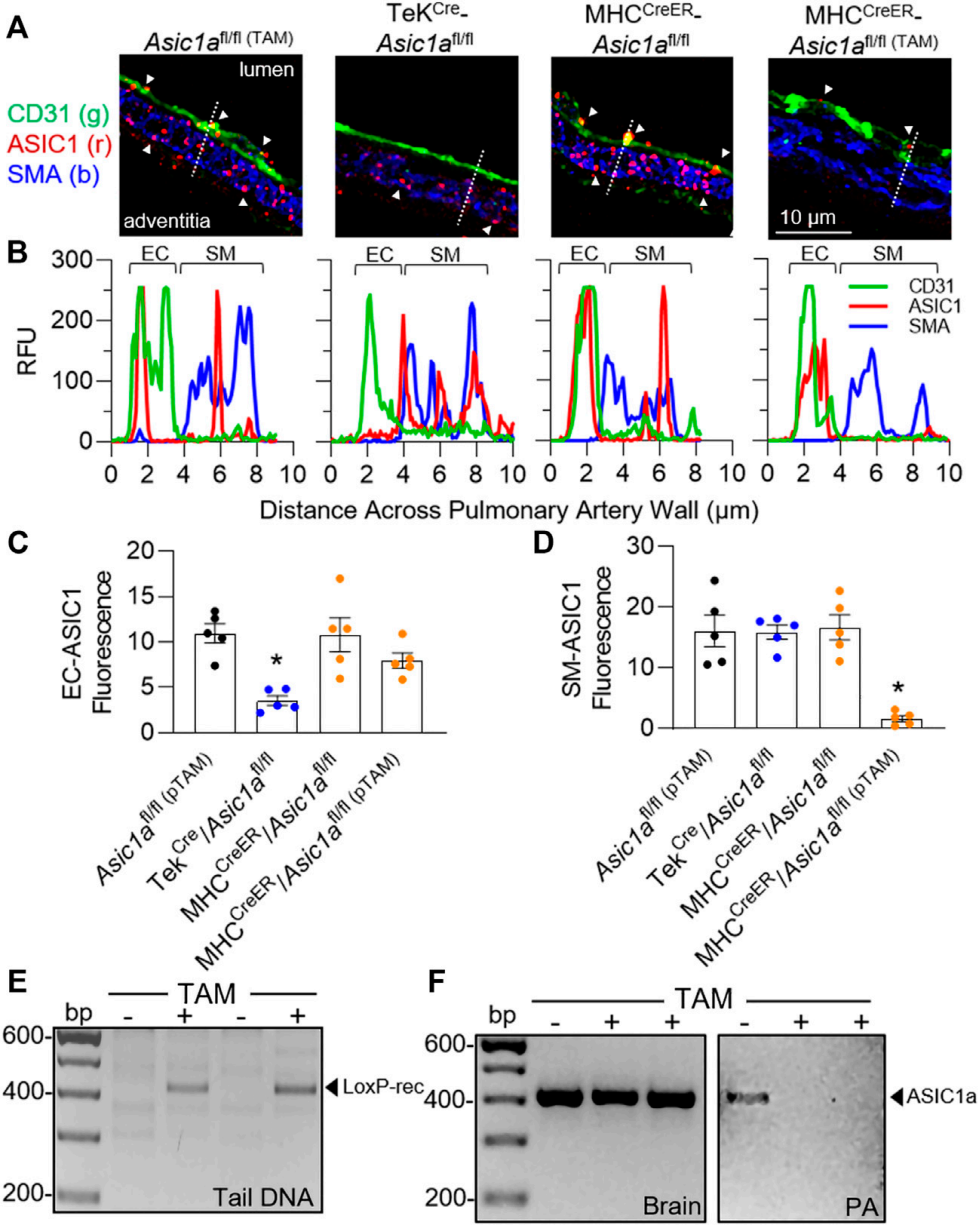
Characterization of transgenic mice. **(A)** Representative immunofluorescence for ASIC1 (red), CD31 (green), and SMA (blue) in pulmonary arteries from lung sections of *Asic1a* ^fl/fl (TAM)^, TekCre-*Asic1a* ^fl/fl^, and MHC^CreER^-*Asic1a* ^fl/fl^ mice treated with vehicle or tamoxifen (TAM). White arrowheads show punctate immunofluorescence of ASIC1 in EC or SM. The dotted line in each image indicates the line profile shown in **(B)** of relative fluorescence units across the artery wall from lumen to adventitia. Summary analysis of ASIC1 expression in either **(C)** EC or **(D)** SM of pulmonary arteries from lung sections. n = 5 animals per group; analyzed by one-way ANOVA and individual groups compared with Šídák’s multiple comparisons tests. **(E)** PCR of tail DNA showing Cre-mediated LoxP recombination and excision of targeted *Asic1a* in the same MHC^CreER^-*Asic1a* ^fl/fl^ mouse before (-) and after (+) tamoxifen (TAM) treatment. **(F)** *Asic1a* mRNA expression in brain tissue and intrapulmonary arteries (PA) in MHC^CreER^-*Asic1a* ^fl/fl^ mice with vehicle (-) or TAM (+). Scanned images of the blots were inverted and adjusted for brightness/contrast.

To determine the specific role of ASIC1a in PAEC and PASMC remodeling in pulmonary hypertension, we generated mice with either EC (Tek^Cre^-*Asic1a* ^fl/fl^) or conditional SM (MHC^CreER^-*Asic1a* ^fl/fl^) specific deletion of *Asic1a.* As demonstrated previously in *Asic1a* ^+/+^ and *Asic1a* ^−/-^ mice (Nitta et al., 2014), ASIC1 was detected as punctate fluorescence within the PASMCs and PAECs from *Asic1a* ^fl/fl^ and MHC^CreER^-*Asic1a* ^fl/fl^ (without TAM-induced Cre recombinase) mice (Figures 3A,B). Line profile through the vessel wall shows that Tek^Cre^-*Asic1a* ^fl/fl^ mice lack expression of ASIC1 in ECs but retain PASMC expression, whereas MHC^CreER^-*Asic1a* ^fl/fl (TAM)^ (with TAM-induced Cre recombinase) lack expression of ASIC1 in PASMCs but retain PAECs expression (Figures 3A–D). Figure 3E shows TAM-induced Cre recombination between *loxP* sites and loss of the intervening genomic sequence (exons 2-3) in tail DNA before and after TAM in the same animal. ASIC1 is highly expressed in the central nervous system and Figure 3F shows that TAM-induced Cre recombinase did not significantly alter *Asic1a* mRNA levels in brain tissue, but there was no detectable expression in isolated pulmonary arteries (PA).

**FIGURE 4:**
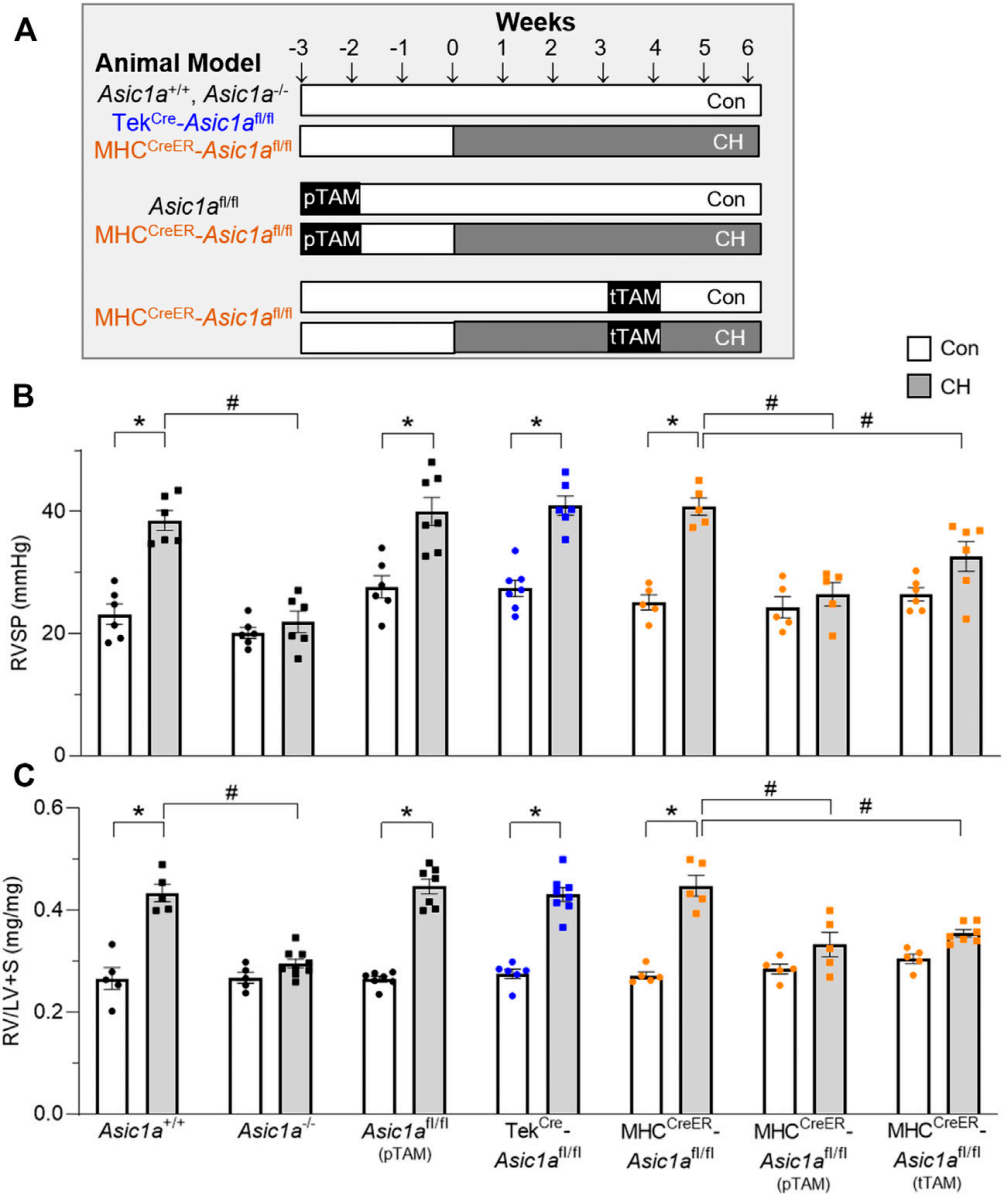
SM-specific knockout of *Asic1a* protects against development of pulmonary hypertension and reverses established hypoxic pulmonary hypertension. **(A)** Experimental design showing treatments of no TAM, preventative (pTAM, administered before CH), and therapeutic (tTAM, administered following established pulmonary hypertension). **(B)** Right ventricular systolic pressure (RVSP, mmHg) and **(C)** Fulton’s Index (RV/LV + S) in *Asic1a* ^+/+^, *Asic1a* ^−/-^, *Asic1a* ^fl/fl (pTAM)^, TekCre-*Asic1a* ^fl/fl^, MHC^CreER^-*Asic1a* ^fl/fl^, MHC^CreER^-*Asic1a* ^fl/fl (pTAM)^, or MHC^CreER^-*Asic1a* ^fl/fl (tTAM)^ mice under control conditions (white bars, circles) or following 6 weeks CH (grey bars, squares). SM-*Asic1a* knockout was induced by treatment with tamoxifen as a preventive (pTAM; before exposure to CH) or therapeutic approach (tTAM; following establishment of CH-induced pulmonary hypertension). n = 5-8/group; analyzed by two-way ANOVA. Significant interactions between the individual groups (*p* < 0.0001 for both RVSP and RV/LV + S) were compared with Šídák’s multiple comparisons tests; *p < 0.05 vs. control; #*p* < 0.05 vs. respective genetic control.

To determine the role of PAEC and PASMC ASIC1a in CH-induced pulmonary hypertension we developed three different CH treatment paradigms represented in Figures 4A, 1) vehicle: *Asic1a* ^+/+^, *Asic1a* ^−/-^, Tek^Cre^-*Asic1a* ^fl/fl^, and MHC^CreER^-*Asic1a* ^fl/fl^ mice were treated with vehicle (corn oil) and 2 weeks later exposed to control or CH for 6 weeks; 2) preventative (pTAM): *Asic1a* ^fl/fl (pTAM)^ and MHC^CreER^-*Asic1a* ^fl/fl (pTAM)^ mice were treated with TAM (5 days) and 2 weeks later exposed to control or CH for 6 weeks; 3) therapeutic (tTAM): MHC^CreER^-*Asic1a* ^fl/fl (tTAM)^ mice were exposed to control or CH for 3 weeks to establish pulmonary hypertension. After 3 weeks CH, mice were treated with TAM (5 days with con/CH exposure) and then continued in con/CH for an additional 2 weeks. Table 3 demonstrates that selective deletion of SM- or EC-*Asic1a* did not significantly alter mean arterial blood pressure or heart rate in conscious mice and is similar to what we have previously recorded in wildtype mice (Detweiler et al., 2019).

**TABLE 3:** Mean arterial blood pressure (MABP) and heart rate in genetically-modified mice treated with or without tamoxifen (pTAM).

	*Asic1a* ^fl/fl^ (4)	Tek^Cre^-*Asic1a* ^fl/fl^ (7)	MHC^CreER^-*Asic1a* ^fl/fl^ (10)	MHC^CreER^-*Asic1a* ^fl/fl (pTAM)^ (8)
MABP (mmHg)	109.2 ± 2.3	109.9 ± 1.5	102.3 ± 1.7	101.7 ± 2.0
Heart Rate (beats/min)	586.6 ± 5.0	553.7 ± 13.1	552.7 ± 5.6	562.9 ± 10.7

Blood pressure and heart rate are 24-h averages taken over 72 h; n’s are indicated in parentheses; analyzed by one-way ANOVA, and individual groups compared with Dunnett’s multiple comparisons tests.

Similar to our previous reports, exposure to CH significantly increased right ventricular systolic pressure (RVSP; Figure 4B) and right heart hypertrophy (Figure 4C) in *Asic1a* ^+/+^, but not *Asic1a* ^−/-^ mice (Nitta et al., 2014). CH led to a similar increase in RVSP and RV hypertrophy in Asic1a^fl/fl (pTAM)^, Tek^Cre^-*Asic1a* ^fl/fl^, and MHC^CreER^-*Asic1a* ^fl/fl^ mice (Figure 4). Along with serving as a control for Tek^Cre^-*Asic1a* ^fl/fl^ and MHC^CreER^-*Asic1a* ^fl/fl^ mice, the *Asic1a* ^fl/fl (pTAM)^ mice also provide evidence that TAM does not affect the development of pulmonary hypertension. Table 4 shows that CH does not affect body mass or heart rate in any of the transgenetic animals. However, the increase in RVSP and RV hypertrophy in *Asic1a* ^fl/fl (pTAM)^, Tek^Cre^-*Asic1a* ^fl/fl^, and MHC^CreER^-*Asic1a* ^fl/fl^ mice is associated with greater RV contractility and workload as indicated by increased dP/dt_max_ and pressure time index, respectively. These data suggest EC-specific deletion of *Asic1* does not contribute to increased RVSP and RV hypertrophy following CH. In contrast, SM-specific deletion of *Asic1a* in MHC^CreER^-*Asic1a* ^fl/fl (pTAM)^ mice by TAM-induced Cre recombinase before CH prevented any CH-induced increases in RVSP, RV hypertrophy, dP/dt_max_, and pressure time index (Figure 4 and Table 3). Additionally, treating MHC^CreER^-*Asic1a* ^fl/fl (tTAM)^ with TAM at week three of the 6-week CH exposure reversed increases in RVSP, RV hypertrophy, dP/dt_max_, and pressure-time index to similar levels as control mice (Figure 4 and Table 3). These data demonstrate that deletion of SM-, but not EC-*Asic1a* both prevents and reverses CH-induced pulmonary hypertension, and RV hypertrophy and dysfunction.

**TABLE 4:** Body mass, heart rate, cardiac contractility, and pressure-time index in anesthetized control and CH genetically-modified mice treated with or without tamoxifen (TAM).

	Group	*Asic1a* ^fl/fl^ ^(pTAM)^	Tek^Cre^-*Asic1a* ^fl/fl^	MHC^CreER^-*Asic1a* ^fl/fl^	MHC^CreER^-*Asic1a* ^fl/fl (pTAM)^	MHC^CreER^-*Asic1a* ^fl/fl (tTAM)^
Body Mass (grams)	Con	23 ± 1	35 ± 5	26 ± 1	26 ± 2	28 ± 1
	CH	23 ± 1	35 ± 2	25 ± 2	28 ± 1	28 ± 1
Heart Rate (beats/min)	Con	475 ± 23	467 ± 16	475 ± 24	411 ± 15	482 ± 29
	CH	493 ± 19	501 ± 34	468 ± 16	423 ± 11	487 ± 27
dP/dt_max_ (mmHg/s)	Con	755 ± 109	1,130 ± 211	704 ± 64	756 ± 188	880 ± 131
	CH	1,238 ± 130 *	2,158 ± 183*	1,333 ± 56*	698 ± 92^#^	908 ± 165
Pressure Time Index (mmHg*s)	Con	1.14 ± 0.09	1.39 ± 0.04	1.40 ± 0.11	1.34 ± 0.18	1.20 ± 0.09
	CH	1.76 ± 0.11 *	1.88 ± 0.09 *	2.15 ± 0.21 *	1.32 ± 0.10^#^	1.54 ± 0.09^#^

The number of animals is indicated in Figure 4; analyzed by two-way ANOVA. Significant interaction between the individual groups (dP/dtmax: *p* = 0.0151; Pressure Time Index: *p* = 0.0249) were compared with Šídák’s multiple comparisons tests; *p < 0.05 control vs. CH; #*p* < 0.05 vs. respective genetic control.

### SM-specific knockout of *Asic1a* attenuates vascular remodeling

**FIGURE 5:**
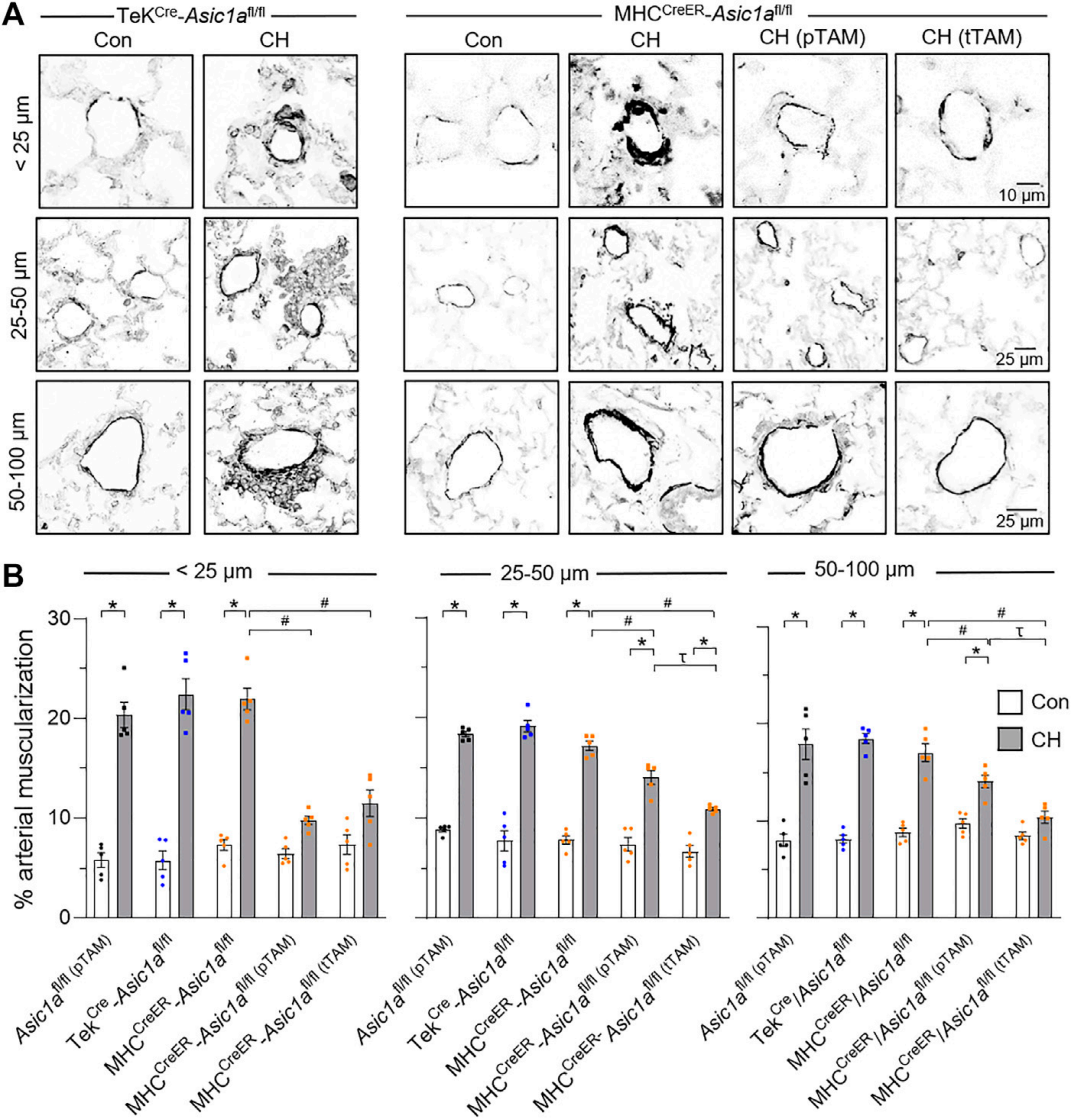
SM ASIC1a contributes to vascular remodeling following CH. **(A)** Representative SMA immunofluorescence (black) images of small pulmonary arteries in lung sections from TekCre-*Asic1a* ^fl/fl^, MHC^CreER^-*Asic1a* ^fl/fl^, MHC^CreER^-*Asic1a* ^fl/fl (pTAM)^, or MHC^CreER^-*Asic1a* ^fl/fl (tTAM)^ mice under control conditions (white bars) or following 6 weeks CH (filled bars). Fluorescence images were digitally inverted to provide better contrast and visibility of immunofluorescence. **(B)** Percent muscularization calculated as percent thresholded SMA area divided by total arterial area based on arterial diameter: <25 μm (n = ∼30 vessels from four animals/group), 25–50 μm (n = ∼100 vessels from four animals/group), or 50–100 μm (n = ∼50 vessels from four animals/group); analyzed by two-way ANOVA. Significant interactions between the individual groups (*p* < 0.0001 for all vessel diameter ranges) compared with Šídák’s multiple comparisons tests. *p < 0.05 vs. control; #*p* < 0.05 vs. (-) TAM; and τ *p* < 0.05 pTAM vs. tTAM.

Vascular remodeling was assessed using immunofluorescence of SM α-actin (SMA) in vessels ranging from <25 μm, 25–50 μm, and 50–100 μm, as shown in Figure 5A. CH caused a significant increase in % muscularization in each artery size from Tek^Cre^-*Asic1a* ^fl/fl^ mice. Some vessels (∼5–10%) from Tek^Cre^-*Asic1a* ^fl/fl^ mice exposed to CH displayed hypercellular lesions projecting outward from the medial and adventitial layers into the adjacent lung parenchyma (Figure 5A). Although this adventitial remodeling was not analyzed as part of the medial thickness, the cells within these areas were observed to express SMA at a lower fluorescence intensity compared to the medial layer, likely representing (myo)fibroblasts. This outward remodeling was not present in wildtype or other transgenic mouse models (Nitta et al., 2014; Detweiler et al., 2019; Sheak et al., 2020), suggesting specific EC deletion of *Asic1a* may facilitate vascular remodeling. CH increased % muscularization in MHC^CreER^-*Asic1a* ^fl/fl^ mice (no TAM-induced Cre recombinase) that was attenuated by both pTAM and tTAM treatments (Figure 5B). SM-specific deletion of *Asic1a* had a greater effect to reduce (neo)muscularization in arteries <25 µm as there was not a significant difference compared to control arteries. Furthermore, deletion of SM-*Asic1a* therapeutically was more effective in reducing arterial muscularization than preventative SM-*Asic1a* deletion. In 50–100 µm arteries from MHC^CreER^-*Asic1a* ^fl/fl (tTAM)^ mice, muscularization was not significantly different between control and CH (Figure 5B).

**FIGURE 6:**
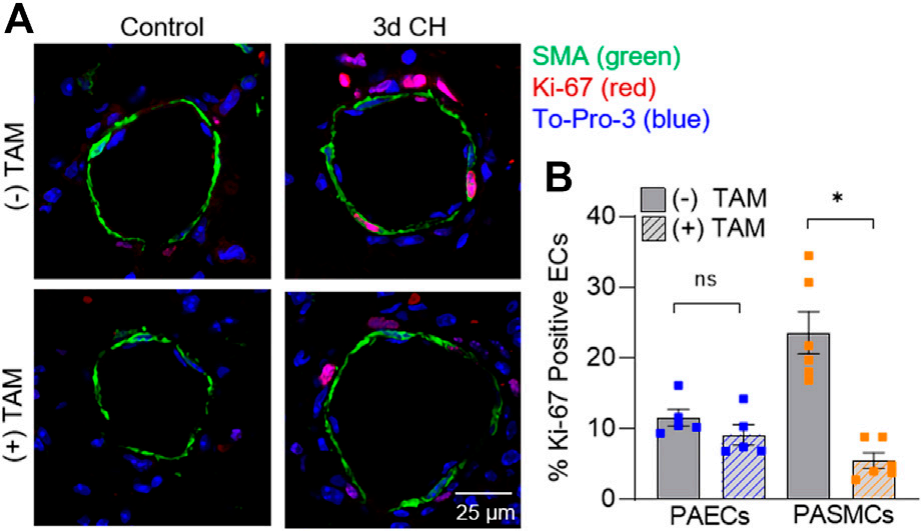
SM ASIC1a contributes to CH-induced PASMC proliferation. **(A)** Representative immunofluorescence images showing Ki-67 (red), α-SMA (green), and Sytox (blue) in small pulmonary arteries (<100 µm) from control and 3 days CH MHC^CreER^-*Asic1a* ^fl/fl^ mice following treatment with vehicle or tamoxifen (TAM). **(B)** Summary data for percent Ki-67 positive cells in endothelial cells (EC, blue) and smooth muscle cells (SMC, orange). Summary data for control mice are not shown since there were no detectable Ki-67 positive cells. n = 75 vessels from five animals (15 vessels each) per group; analyzed by unpaired *t*-test; #*p* < 0.05 vs. (-) TAM 3-days CH; ns = not significant.

PAEC and PASMC proliferation in MHC^CreER^-*Asic1a* ^fl/fl^ mice was additionally evaluated using immunofluorescence to identify Ki-67 positive cells (Figure 6). Deletion of SM-*Asic1a* did not significantly affect the % of positive Ki-67 nuclei in PAECs, but significantly reduced the percent of proliferating PASMCs (Figure 6B). Together these data show that SM-*Asic1a* contributes to CH-induced vascular remodeling by contributing to both muscularization and PASMC proliferation.

### ASIC1 contributes to PASMC migration and proliferation

**FIGURE 7:**
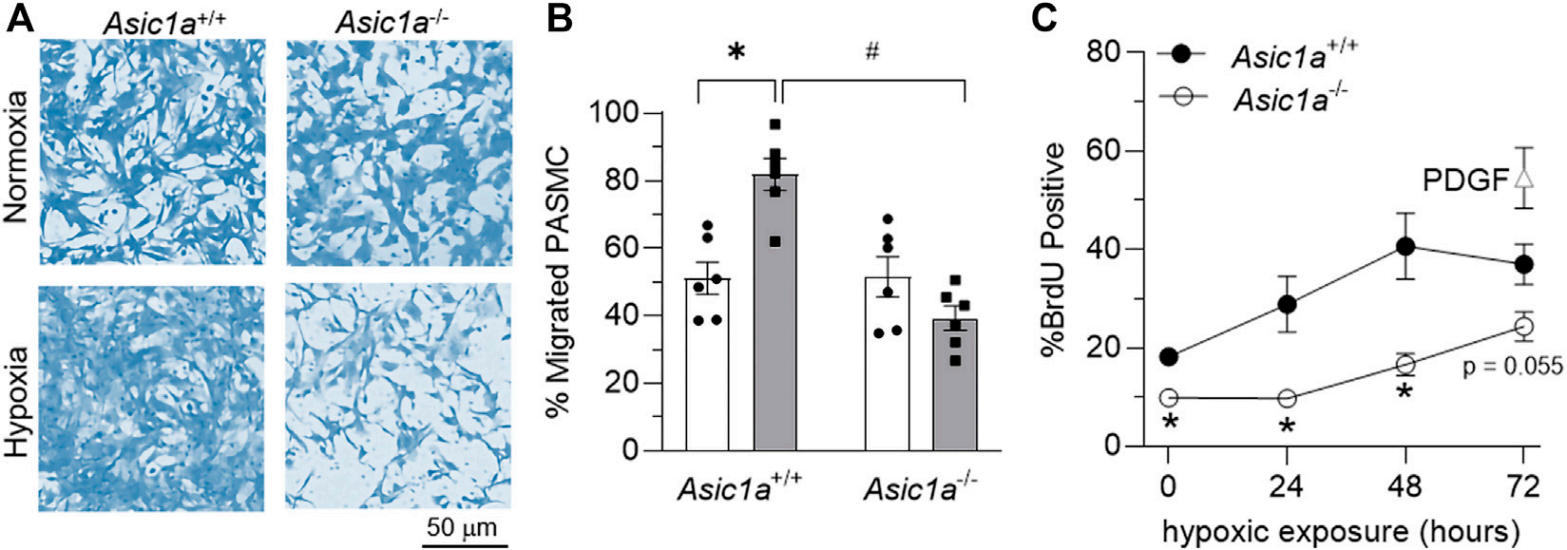
ASIC1a contributes to mPASMC migration and proliferation. **(A)** Representative brightfield images of coomassie-stained mPASMC and **(B)** summary data for % of migrated mPASMC from *Asic1a* ^+/+^ and *Asic1a* ^−/-^ mice exposed to normoxia (5% CO_2_, 95% air; orange circles) or hypoxia (5% CO_2_, 2% O_2_; blue squares) for 24 h n = 6 animals/group; *p < 0.05 vs. hypoxia and #*p* < 0.05 *Asic1a* ^+/+^; analyzed by two-way ANOVA. Significant interactions between the individual groups (*p* = 0.0003) compared using Šídák’s multiple comparisons tests. **(C)** Flow cytometry analysis of percent of mPASMC from *Asic1a* ^+/+^ and *Asic1a* ^−/-^ mice with BrdU incorporation under normoxia (0 h; 5% CO_2_, 95% air) or hypoxia (5% CO_2_, 2% O_2_) for 24, 48, or 72 h. Normoxic mPASMC from *Asic1a* ^+/+^ were incubated with PDGF-BB (20 ng/ml) for 72 h as a positive control. n = five to eight animals/group, analyzed by unpaired t-tests at each time point, *p < 0.05.

PASMCs were exposed to *in vitro* hypoxia, followed by assessing migration and proliferation via transwell assays and flow cytometry for BrdU-positive cells, respectively. Hypoxia significantly increased the percent of migrating mPASMCs from *Asic1a* ^+/+^, but not *Asic1a* ^−/-^ mice (Figures 7A,B). Proliferation, as assessed by BrdU incorporation, was significantly less in mPASMCs from *Asic1a* ^−/-^ mice under normoxia and following exposure to 24 and 48 h hypoxia (Figure 7C).

**FIGURE 8:**
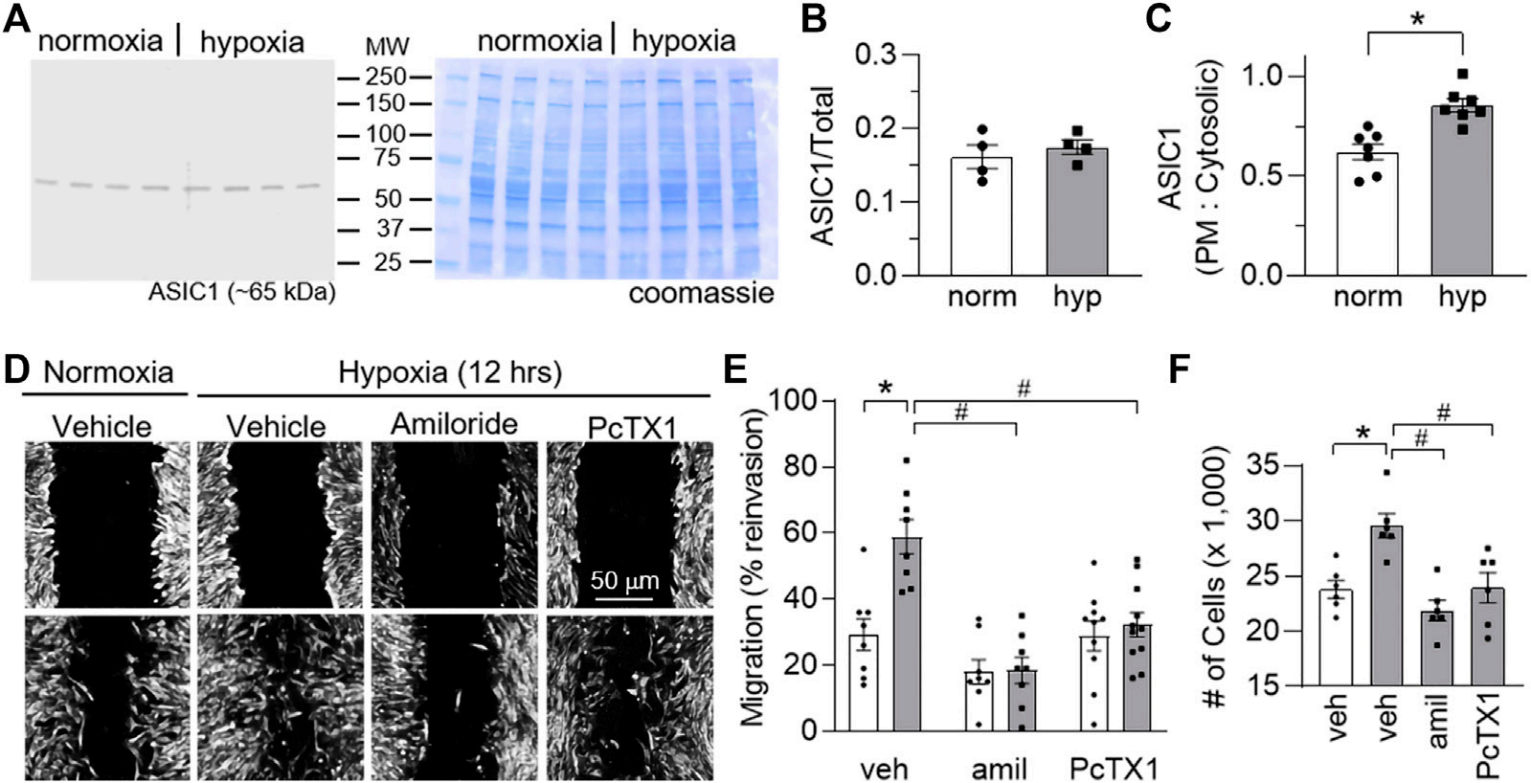
ASIC1 contributes to human PASMC migration and proliferation. **(A)** Representative Western blots of ASIC1 (predicted ∼65 kDa) and corresponding coomassie-stained blot and **(B)** summary data showing ASIC1 expression (normalized to entire lane on coomassie-stained blot) in human PASMC (hPASMC) exposed to normoxia (norm, 5% CO_2_, 95% air; white bars/circles) or 12 h hypoxia (hyp, 5% CO_2_, 2% O_2_; grey bars/squares). Scanned images of the film were converted to greyscale and adjusted for brightness/contrast. **(C)** Summary data showing Western blot analysis for biotinylated (plasma membrane) and cytosolic ASIC1 protein expression in hPASMC exposed to normoxia or hypoxia. **(D)** Brightfield images of hPASMC immediately following the scratch (baseline time 0; top row of images) or 12 h post-exposure (bottom row of images) to normoxia (5% CO_2_, 95% air; orange circles) or hypoxia (5% CO_2_, 2% O_2_; blue squares) and **(E)** summary data showing percent reinvasion of hPASMC into the wounded area in the presence of vehicle, amiloride or PcTX1. *n* = 8-11/group; analyzed by two-way ANOVA. Significant interactions between the individual groups (*p* = 0.0032) were compared using Šídák’s multiple comparisons tests. **(F)** increase in the number of cells after 24 h of normoxia or hypoxia in the presence of vehicle, amiloride, or PcTX1. hPASMC were initially plated at a density of 15,000 cells/well. *n* = 6/group; analyzed by one-way ANOVA and individual groups compared using Šídák’s multiple comparisons tests. *p < 0.05 vs. normoxic group; #*p* < 0.05 vs. corresponding vehicle group.

To determine if these findings translate to human PASMCs (hPASMCs) we assessed the effects of hypoxia on ASIC1 expression, migration, and proliferation in hPASMCs in the absence or presence of ASIC1 inhibition. Although 12 h hypoxia did not alter the total expression of ASIC1 (Figures 8A,B), it significantly increased the plasma membrane (cell surface) compared to cytosolic expression (Figure 8C). This is consistent with previous studies in 4 weeks CH-exposed rats where total expression of ASIC1 is unchanged but hypoxia causes a subcellular translocation of ASIC1 to the plasma membrane (Herbert et al., 2016, 2018). The percent reinvasion of hPASMCs was greater following 12 h hypoxia compared to normoxia and this was prevented by pre-treatment with amiloride or PcTX1 (Figures 8D,E). Hypoxia-induced proliferation was additionally blocked by amiloride and PcTX1 in hPASMCs (Figure 8F). Taken together, these data suggest ASIC1 involvement in hypoxia-mediated hPASMC proliferation and migration.

## Discussion

Our laboratory has previously demonstrated that ASIC1a contributes to the development of CH-induced pulmonary hypertension by contributing to enhanced agonist-induced vasoconstriction and vascular remodeling (Nitta et al., 2014). In the pulmonary circulation, ASIC1 is expressed in both PASMCs and PAECs and the goal of the current study was to determine if there is a differential contribution of EC and SM ASIC1 to the development of pulmonary hypertension. We found that specific deletion of EC-*Asic1a* did not affect the development of pulmonary hypertension; whereas deletion of SM-*Asic1a* prevented CH-induced pulmonary hypertension and reduced medial vascular remodeling. This reduction in remodeling was associated with decreased proliferation and migration of PASMCs from *Asic1a* ^−/-^ mice. We further demonstrate that deletion of SM-*Asic1a* in mice with established pulmonary hypertension effectively reversed increases in RVSP, RV hypertrophy, and vascular remodeling, signifying ASIC1 as a potential therapeutic target for pulmonary hypertension.

In the pulmonary circulation, ASIC1 is activated in response to various vasoactive factors (endothelin-1, UTP) and alveolar hypoxia resulting in PASMC Ca^2+^ influx and pulmonary arterial constriction (Jernigan et al., 2012; Nitta et al., 2014). Inhibition of ASIC1 or *Asic1a* gene deletion abolishes the enhanced agonist-induced vasoconstriction following CH. The activation of ASIC1a in PASMCs following stimulation of G-protein coupled receptors appears to be independent of pH changes. Although we do not know the exact mechanism leading to non-proton activation of ASIC1, our previous work demonstrates ASIC1a is activated secondary to store-depletion of the sarcoplasmic reticulum, a mechanism referred to as store-operated Ca^2+^ entry (Jernigan et al., 2009, 2012). We have also demonstrated that ASIC1 contributes to acute hypoxic pulmonary vasoconstriction (Nitta et al., 2014) and the persistent PASMC membrane depolarization following CH exposure (Jernigan et al., 2021). Despite the requirement for ASIC1 in the development of pulmonary hypertension, this response is not dependent on an increase in total ASIC1 protein expression. Rather hypoxia causes subcellular relocalization of ASIC1 to the plasma membrane (Nitta et al., 2014; Herbert et al., 2018), a response that occurs in hPASMC as early as 12 h of hypoxic exposure (Figure 8). Furthermore, we have recently demonstrated that primary-cultures of PASMC from pulmonary hypertensive animals show a shift in cellular metabolism that promotes glycolysis and lactic acid fermentation leading to extracellular acidification (Tuineau et al., 2022). Further research is necessary to determine the importance of this pH shift to activate ASIC1 in pulmonary hypertension.

Although *Asic1a* ^−/−^ mice are also protected from CH-induced vascular remodeling and right ventricular hypertrophy (Nitta et al., 2014), it is unclear if ASIC1a is directly involved in the remodeling process or whether ASIC1a indirectly promotes remodeling by increasing vasoconstriction and pulmonary vascular resistance. The current findings that ASIC1a contributes to hypoxia-induced PASMC proliferation and migration support a direct contribution of ASIC1a to vascular remodeling. These data corroborate several studies showing that ASIC1, expressed in a variety of cancers, plays a role in regulating multiple malignant processes including proliferation, migration, epithelial-mesenchymal transition, and cell cycle progression (Kapoor et al., 2009; Rooj et al., 2012; Jin et al., 2015; Wu et al., 2017; Zhu et al., 2017; Chen et al., 2018; Ding et al., 2021; Zhu et al., 2021). Conversely, prevention of right ventricular hypertrophy following deletion of SM-*Asic1a* suggests cardiomyocyte remodeling in this mouse model of hypoxic pulmonary hypertension largely occurs due to the role of ASIC1a to increase pulmonary vascular resistance.

Early studies proposed that the initial increase in pulmonary vascular resistance in response to hypoxic exposure is largely due to hypoxic pulmonary vasoconstriction; whereas the structural changes in the pulmonary vascular bed following sustained exposure to hypoxia are the major determinant of elevated vascular resistance with disease progression (Sime et al., 1971; Lockhart et al., 1976; Fried et al., 1983). Interestingly, however, studies show no active PASMC proliferation in end-stage lung tissue from idiopathic and hereditary pulmonary arterial hypertensive patients (Majka et al., 2008) suggesting active proliferation occurs early in the disease process as we observed in mice. Although the degree of CH-induced pulmonary hypertension, right ventricular hypertrophy, and pulmonary vascular remodeling (mainly medial muscularization) in mice is modest compared to some other species, the same cellular processes seem to be involved and genetically modified mice allow us to investigate the function of specific proteins in pulmonary hypertension. Furthermore, our data is consistent with other studies, showing actively proliferating PASMCs and PAECs within the first 3–5 days of hypoxic exposure that subsides by 4 weeks CH (Meyrick & Reid, 1979; Quinlan et al., 2000; Paddenberg et al., 2007; Nozik-Grayck et al., 2008; Bierer et al., 2011). This increase in proliferating vascular cells at 3-days CH corresponds with a significant decrease in lung SM MHC expression suggesting PASMC phenotypic switching–the transition from the quiescent contractile to the proliferative synthetic phenotype (Owens, 2007). Previous research in our laboratory demonstrates that the Ca^2+^/calcineurin-dependent transcription factor known as nuclear factor of activated T cells isoform-3 (NFATc3) is required for CH-induced pulmonary arterial remodeling. This process involves an initial proliferation of PASMC (dedifferentiation) followed by differentiation (upregulation of differentiation marker soluble guanylyl cyclase α1) and hypertrophy of PASMC (upregulation of SMA) (de Frutos et al., 2007, 2009; Bierer et al., 2011). Importantly, we showed that ASIC1-dependent Ca^2+^ influx stimulates NFATc3 activation following 5-days CH, providing an essential link between activation of ASIC1a and transcriptional regulation of PASMC phenotypic transformation (Gonzalez Bosc et al., 2016). Whether ASIC1a regulates other transcription factors essential to pulmonary vascular remodeling, like FOXM1 (Dai J et al., 2018; Dai Z et al., 2018), requires further investigation.

PASMCs play a central role in vascular remodeling due to the remarkable ability to dynamically modulate their phenotype to ensure contractile and synthetic functions (Wang et al., 2015; Roostalu et al., 2018). TAM-induced deletion of SM-*Asic1a* normalized RVSP in CH-exposed MHC^CreER^-*Asic1a* ^fl/fl^ mice to near control levels in both preventative and therapeutic protocols. Although knockdown of SM-*Asic1a* effectively eliminated remodeling in intra-acinar vessels (<25 µm), there was still a considerable degree of muscularization in small arteries (25–100 µm). This could signify a different contribution of ASIC1a to hypertrophy, hyperplasia (proliferation), and migration and how these remodeling processes differ in pre-capillary (intra-acinar) versus small arteries. Hypertrophy plays a large role in overall medial thickening and we have previously shown that PASMCs from *Asic1a* ^−/-^ mice do not exhibit CH-induced hypertrophy (Jernigan et al., 2021). Using [^3^H]-thymidine uptake as a marker of proliferation, previous studies in hypoxic-exposed rats have demonstrated that SM cell proliferation doubles in large pulmonary arteries while intra-acinar arteries do not show evidence of [3H]-thymidine uptake (Meyrick & Reid, 1979). Rather, the remodeling of the intra-acinar arteries involves the appearance of cells expressing SM-specific markers in normally non-muscular vessels. This is thought to be mediated mainly by distal migration of nonproliferative SM cells and differentiation of existing precursor SM cells and/or pericytes (Meyrick & Reid, 1979). ASIC1a may contribute to migration more than proliferation, as suggested by the *in vitro* assay in which BrdU incorporation at 72 h hypoxia was not statistically different between PASMCs from *Asic1a* ^+/+^ and *Asic1a* ^−/-^ mice (*p* = 0.055; Figure 7).

Mice develop moderate PH and right ventricular hypertrophy when exposed to CH and this is associated with modest pulmonary vascular remodeling (mainly medial muscularization). As such, it is worth noting the limitations of assessing the role of PASMC ASIC1a in CH-induced remodeling in MHC^CreER^-*Asic1a* ^fl/fl^ mice. First, other SM precursors, pericytes, and/or (myo)fibroblasts contribute to vascular remodeling following CH. These “dedifferentiated” SM-like cells lack expression of MHC, which drives *Asic1a* deletion in MHC^CreER^-*Asic1a* ^fl/fl^ mice. This may explain why remodeling is more effectively inhibited in global *Asic1a* ^−/-^ (Nitta et al., 2014) compared to MHC^CreER^-*Asic1a* ^fl/fl^ mice. Indeed, evaluation of SM cell profile using lineage tracing shows very few mature medial MYH11^+^ SM cells within the cell population of atherosclerotic lesions (Chappell et al., 2016; Jacobsen et al., 2017; Misra et al., 2018). Second, deletion of SM-*Asic1a* therapeutically (tTAM) was more effective in reducing arterial muscularization than preventative SM-*Asic1a* deletion (pTAM). Although MHC expression is decreased following 3 days of CH when cells are proliferating, MHC and SMA (de Frutos et al., 2007) expression are upregulated at 7 days of CH exposure. This suggests the newly proliferated cells are transitioning to contractile SM leading to more PASMC in the medial layer and a greater number of PASMC to target with MHC-driven gene recombinase. Importantly, our data demonstrate that deletion of SM-*Asic1a* in established pulmonary hypertension not only halts remodeling but leads to reversal of the remodeling process. Further studies are necessary to determine the mechanism of this reversal.

Interactions between PASMCs and PAECs are essential for the maintenance of PASMC phenotype, and PAEC dysfunction in pulmonary hypertension leads to proliferation and migration of resident vascular cells and induces a PASMC phenotypic switch (Humbert et al., 2008). Although the functional role of ASIC1a in PAECs is unknown; mesenteric endothelial cell ASIC1a contributes to endothelial-dependent vasodilation through activation of intermediate- and small-conductance Ca^2+^-activated K^+^ channels (Garcia et al., 2018). Based on these studies, we anticipated selective loss of EC-*Asic1a* would lead to endothelial dysfunction and exacerbate pulmonary hypertension. On the contrary, Tek^Cre^-*Asic1a* ^fl/fl^ mice develop pulmonary hypertension comparable to *Asic1a* ^+/+^ (or *Asic1a* ^fl/fl^) mice. Despite a similar RVSP, however, we observed that remodeled pulmonary arteries from Tek^Cre^-*Asic1a* ^fl/fl^ mice had a more advanced outward remodeling than typically observed in mice. Similar prominent outward remodeling has been noted in rats exposed to SU5416/CH (Jernigan et al., 2017), cows with Brisket’s disease [hypoxic pulmonary hypertension in cattle residing at high altitudes] (Davie et al., 2006; Newman et al., 2011), and patients with pulmonary arterial hypertension (Stacher et al., 2012). As with human lung samples, we were unable to quantitate the outward adventitial remodeling due to methodological limitations. First, the pronounced outward remodeling was only observed in ∼5–10% of arteries analyzed. Although these cells express SMA, it is sparse and lower intensity than PASMCs in the medial layer, and migration into the parenchyma makes the precise boundaries difficult to demark. Furthermore, this adventitial remodeling that occurred in a small proportion of arteries is likely not sufficient to raise RVSP since the increase in vessel wall thickness predominantly occurred in an outward direction without encroachment on the lumen. Therefore, although the overall effect on RVSP and RV hypertrophy was minimal with the loss of EC-*Asic1a*, we currently cannot discount a possible role of EC ASIC1a to mitigate pulmonary vascular medial remodeling.

## Data Availability

The raw data supporting the conclusions of this article will be made available by the authors, without undue reservation.
